# CLIP-GENE: a web service of the condition specific context-laid integrative analysis for gene prioritization in mouse TF knockout experiments

**DOI:** 10.1186/s13062-016-0158-x

**Published:** 2016-10-24

**Authors:** Benjamin Hur, Sangsoo Lim, Heejoon Chae, Seokjun Seo, Sunwon Lee, Jaewoo Kang, Sun Kim

**Affiliations:** 1 Interdisciplinary Program in Bioinformatics, Seoul National University, Daehak-dong, Seoul, 151-742 Korea; 2 Department of Computer Science, School of Informatics and Computing, Indiana University, 150 S. Woodlawn Avenue, Bloomington, 47404 USA; 3 Department of Computer Science and Engineering, Seoul National University, Daehak-dong, 151-742 Seoul, Korea; 4 Department of Computer Science and Engineering, Korea University, Seoul, Korea; 5 Bioinformatics Institute, Seoul National University, Daehak-dong, Seoul, 151-742 Korea

**Keywords:** Knockout mouse, Gene prioritization, Gene selection, Web tool

## Abstract

**Motivation:**

Transcriptome data from the gene knockout experiment in mouse is widely used to investigate functions of genes and relationship to phenotypes. When a gene is knocked out, it is important to identify which genes are affected by the knockout gene. Existing methods, including differentially expressed gene (DEG) methods, can be used for the analysis. However, existing methods require cutoff values to select candidate genes, which can produce either too many false positives or false negatives. This hurdle can be addressed either by improving the accuracy of gene selection or by providing a method to rank candidate genes effectively, or both. Prioritization of candidate genes should consider the goals or context of the knockout experiment. As of now, there are no tools designed for both selecting and prioritizing genes from the mouse knockout data. Hence, the necessity of a new tool arises.

**Results:**

In this study, we present CLIP-GENE, a web service that selects gene markers by utilizing differentially expressed genes, mouse transcription factor (TF) network, and single nucleotide variant information. Then, protein-protein interaction network and literature information are utilized to find genes that are relevant to the phenotypic differences. One of the novel features is to allow researchers to specify their contexts or hypotheses in a set of keywords to rank genes according to the contexts that the user specify. We believe that CLIP-GENE will be useful in characterizing functions of TFs in mouse experiments.

**Availability:**

http://epigenomics.snu.ac.kr/CLIP-GENE

**Reviewers:**

This article was reviewed by Dr. Lee and Dr. Pongor.

**Electronic supplementary material:**

The online version of this article (doi:10.1186/s13062-016-0158-x) contains supplementary material, which is available to authorized users.

## Introduction

Measuring RNA-seq data from the knockout mice experiment is widely used to characterize the function of a gene at the in vivo level. By taking the advantage of high-resolution data, the combination of RNA-seq and the knockout mice experiment have demonstrated its utility to determine genes that can explain the phenotypic differences between knockout and wild type mice [1]. Analyzing differentially expressed genes (DEGs) is one of the most widely used method to explain the altered patterns of gene expression between wild type and knockout mice. However, the DEG method has several limitations in explaining the relationship between the alteration of gene expression and the knockout gene. First, the number of genes that are estimated as DEGs are typically large and varies due to the diversity of the underlying models, such as options, thresholds, and p-values. Thus it is challenging to focus on genes that are related to the phenotype [2], even if the method provides statistical scores to prioritize genes. Furthermore, linking the phenotypic difference with identified DEGs lacks in logical explanation since DEG methods do not consider the complex interactions among genes. For these reasons, it is difficult to select genes that are related to the phenotypic differences in samples.

To overcome the limitations of the DEG methods, studies have suggested several integrative analysis techniques that utilize additional information to effectively identify genes that are related to the phenotypic differences. Integrative analysis techniques typically utilize networks such as gene regulatory network (GRN), protein-protein interaction (PPI), or pathway information to determine genes that are related to the phenotypic differences. GRN is shown to be useful in determining the regulatory role of certain genes by using various expression data [3–5]. PPI and pathway information are both networks from the documented biological knowledge to consider gene-gene relationships [6]. In addition, the high throughput sequencing data can be used to exclude genes that may be expressed differentially due to the genetic differences in different samples by identifying single nucleotide variants (SNVs). This technique is particularly useful with small number of samples to identify genes related to the actual phenotypic differences regardless of genetic differences [7]. Although these methods are effective in narrowing down to the actual candidate genes to a few hundreds, researchers need more information to prioritize genes that are more relevant to the phenotypic differences.

In the past few years, many studies have proposed methods to prioritize genes from a large pool of candidates [8] by utilizing various data sources such as gene ontology, PPI, signaling pathways, literature search, and more. However, it is known that the heterogeneous data sources cause difficulties to integrate multiple data sources. The complexities among data sources cause compatibility issues and makes it difficult to understand the relationship between the input data and the final prioritized results since it lacks in logical ‘explanation’ [8]. Thus it is necessary to integrate these heterogeneous data sources consistently in a single framework.

## Motivation

Even though previous studies proposed many useful computational methods to prioritize genes, there should be more efforts to design, implement, and deliver usable software packages for researchers. The motivations of our study are as follows.

First, most existing gene prioritization tools are not appropriate for the condition specific data such as mice knockout data. When a certain gene is knocked out, researchers have specific hypotheses that are related to the observed phenotypic differences. Thus, to select genes that are related to phenotypic differences, it is important to not only consider gene expression alteration but also to prioritize genes with the researcher’s interest. Without considering the condition or the goal of experiment, prioritization results would lack explanation on ‘how and why genes are ranked’. The best strategy is to provide information about the conditions of the experiment or specific hypothesis that the user has. When the user provides such information, genes can be prioritized by consulting the literature database. Therefore, it is necessary to perform an integrative analysis of transcriptome data and literature data for the condition specific gene selection and prioritization.

Second, complex relationships among genes should be considered in order to selected and prioritize genes that are related to the phenotype. Therefore, networks such as GRN and PPI are useful in explaining alteration among genes by considering gene-gene and regulatory relationships. Many knockout experiments have investigated transcription factors (TFs) that could result in the phenotypic differences by analyzing the GRN [9–12]. Thus, considering GRN (to be specific, TF network) is essential to characterize the roles of TFs from knockout data. In addition to TF networks, PPI networks also assist in explaining expression alteration among genes since PPI networks consist more entities than other networks such as TF networks and biological pathway networks. Since we need to use both TF and PPI networks, an issue is how to utilize two different networks in a single computational framework. Our approach uses TF network to select candidate genes from TF knockout experiment and uses PPI to prioritize candidate genes in combination of the literature information in a condition specific manner.

Third, existing computational methods for prioritizing genes are not designed for mouse knockout data. Only 3 among 27 tools (listed in Gene Prioritization Portal [13]) are designed for the mouse data [14–16]. However, we think that these tools are generally not applicable to evaluate RNA-seq data of knockout experiments. For example, even though PINTA [16] and GeneFriends [14] can prioritize genes based on the concept of the guilt-by-association or network analysis, these tools require a pre-selected gene list of a certain size: up to 200 genes in PINTA and up to 500 genes in GeneFriends. Both tools are not applicable when the number of genes are large, such as DEG results. Although use of a stringent cutoff value can reduce the number of candidate genes that can be used for aforementioned tools, there may be too many false negatives. Therefore, the requirement of a pre-selected gene list in PINTA and GeneFriends is not easy to be resolved. In addition, PINTA is designed for microarray data and prioritizes genes by referring the expression profiles of its neighbors from the PPI network, but it does not consider the influence of the knockout gene. Likewise, GeneFriends prioritizes genes by considering co-expression of other genes but does not reflect the effect of the knockout gene. Another tool, Endeavor [15], is able to prioritize genes from a large number of gene list that does not require pre-selection from gene list. However, Endeavor requires a gene list from prior knowledge as a training dataset, and it is designed to select disease related genes rather than knockout related genes. By considering all issues, we introduce CLIP-GENE (Context Laid Integrative analysis to Prioritize genes), a web based tool that takes a DEG list as input and uses TF network and SNV information to narrow down candidate genes and prioritizes genes with PPI information and literature information. In particular, CLIP-GENE allows researchers to specify the context of the experiment as a set of keywords input to a biomedical entity search tool (BEST) [17].

## Methods

### Workflow of CLIP-GENE

**Fig. 1:**
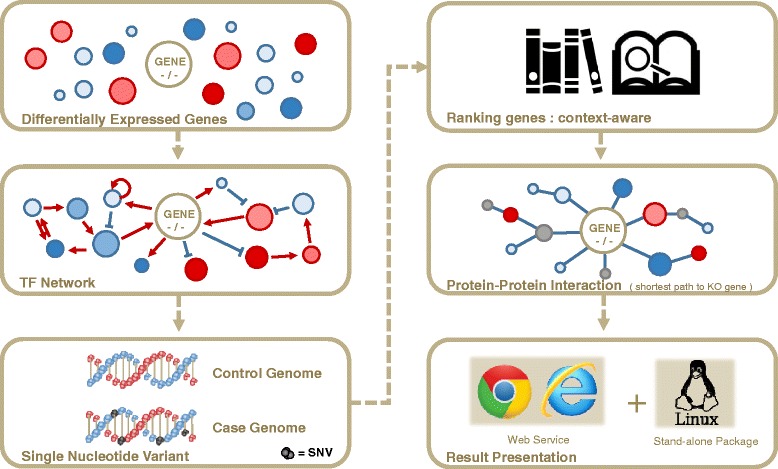
A Workflow of CLIP-GENE. CLIP-GENE prioritize user-interested genes that are relevant to phenotypic/functional differences of knockout mice data. CLIP-GENE takes DEG as input and filter out genes by using TF network and SNV information. Then prioritize these genes by using BEST and PPI information

CLIP-GENE selects and prioritizes genes in two major steps. For the selection step, TF network and SNV information are used to select candidate genes that are affected by the knockout gene as well as expressed differentially between wild type and knockout mice. For prioritization, BEST and PPI information are used to prioritize genes according to the researcher’s context or hypothesis. With the assistance of a literature search tool BEST, it allows to specify certain context or hypothesis with a set of keywords by user that is expected from the data. Afterwards, PPI is used to consider the gene-gene relationship between the candidate genes and the knockout gene. Workflow of CLIP-GENE is illustrated in Fig. 1. Details of each step are described below.

### Step 1: selection of candidate genes

CLIP-GENE takes a DEG list from the knockout experiment and investigates the regulatory role of the DEGs by referring to TF network. The methods for DEG selection and the TF network construction are described in Materials section.

**Step 1-1: selecting candidate DEGs using TF network.** CLIP-GENE takes a list of DEGs as input and uses them as initial candidates. Then, by referring to the mouse TF network that was constructed using 150 mice expression profiles, DEGs that do not affect other DEGs or DEGs that are not affected by the knockout gene are excluded. This step is performed to focus on the relationship between the regulator and its target genes that are significantly altered.

**Step 1-2: removing DEGs caused by genetic difference.** After CLIP-GENE selects candidate DEGs that takes a part in the regulatory role, SNV information is used to filter out DEGs that might be caused by the genetic differences rather than the influence of the knockout gene. It is well known that even if the inbred mice are raised in a controlled environment, genetic differences are likely to be present [18]. If we can perform a large number of RNA-seq experiments, it is possible to screen genes that may be expressed differentially due to the genetic difference. However, it is not practical to perform such a large number of RNA-seq experiments that is enough to remove such genes. To compensate the low statistical power of the typical RNA-seq data, candidate genes with over than a certain rate of SNVs in the knockout mice are discarded [7].

### Step 2: prioritizing genes with the user context & PPI

Candidate genes selected in Step 1 are ranked in terms of the relevance to the phenotype in two different criteria: the user specified context and the PPI information.

**Step 2-1: rank genes with user’s interest** CLIP-GENE users can specify their hypothesis for the knockout data as ‘context’ in a set of keywords. Specifically, context means a set of subjective words that describe the user’s interest such as ‘expected biological function when the gene is knockout’ or ‘known function of the knockout gene’. For example, a context for Gata3 knockout data can be described as ‘Immune response’, ‘Cell signaling’, or ‘Inflammatory response’ [19, 20]. Then genes that are related to the user-specified keywords can be determined by looking for the relevance between keywords since certain keywords are documented in the literature in relation to a certain gene. Thus this can be viewed as a process to find keyword-keyword relationship and keyword-gene relationship to prioritize genes.

In order to find the relevance between two different keywords, literature search systems based on the named entity recognition (NER) are known to be effective [21]. For CLIP-GENE, BEST [17] is used to find the relevance between knockout gene and candidate genes as well as the relationship between candidate genes and the user given context. With the user specified keywords, BEST computes relevance between any pair of keywords from PubMed and returns a relevance score of genes with ranks. Once the relevance score of ‘context to candidate gene’ and ‘knockout gene to candidate gene’ is calculated, the maximum of them is used to represent how the candidate gene is relevant to the user’s interest or the knockout gene. As a result, a candidate gene with a higher relevance score is ranked with higher priority.

**Fig. 2:**
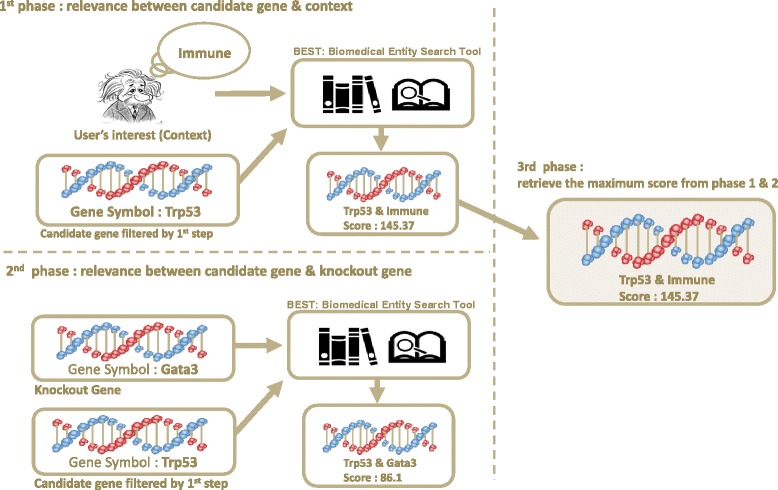
Prioritization step 1: Prioritizing genes with Biomedical Entity Search Tool (BEST). BEST is utilized to find the relevance between knockout gene and candidate gene as well as the relationship between candidate gene and given context. Then CLIP-GENE retrieves the maximum score to represent that the candidate gene is highly relevant to the user’s interest or knockout gene. As a result, candidate gene with higher relevance score is ranked with high priority

**Fig. 3:**
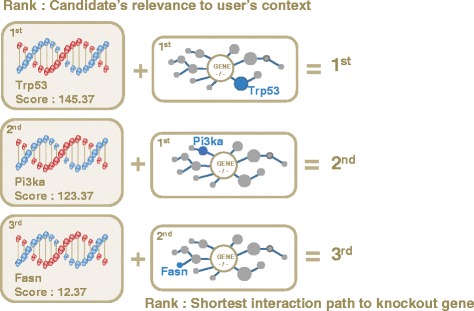
Prioritization step 2: Prioritizing genes with Biomedical Entity Search Tool (BEST) and PPI information. CLIP-GENE summarizes ranks from step 1 and PPI shortest path by using Borda count

**Step 2-2: rank genes using PPI** PPI information is used to rank candidates by computing the shortest interaction path to the knockout gene on the STRING PPI network [22]. Candidates that have shorter interaction path to the knockout gene are considered to be more relevant to the phenotypic/functional difference, hence they are ranked with a higher priority. Finally, CLIP-GENE summarizes candidates with ranks by combining the BEST and PPI information with unweighted Borda count [23]. Figures 2 and 3 describes the overview of gene prioritization.

## Results

### Evaluation of CLIP-GENE

For the performance evaluation, we used datasets that come with publications reporting which genes are relevant to the functional difference when the gene is silenced. These genes are used as true positives to measure the precision, recall, and F-measure in terms of genes reported in the publications for data sets, GSE47851 [19], GSE54932 [24], and GSE53398 [25]. CLIP-GENE was compared with methods and tools that can be used for RNA-seq mouse data. In this study we compared with DEG method (DEG), integrative analysis method (IA) [7], and GeneFriends [14] in terms of the predictive power. In addition, since the user can specify context with a set of keywords, the performance depends on the context that the user provides. In this experiment, we used the four different sets of keywords as context. To compare the predictive power, we designated the best case and the worst case in terms of the number of genes reproduced by CLIP-GENE. In addition, as BEST investigates the relationship between two given keywords by referring the abstract from PubMed, we chose keywords that were not mentioned in the abstract of the corresponding publications. This process is done to make sure that BEST did not consider the keywords from the publication that generated the data while calculating the relevance score.

Dataset GSE47851 is from a Gata3 knockout mouse study that reported 25 genes were relevant to the functional difference between the wild type and the knockout. For the performance evaluation, we used four different contexts: ‘Inflammatory response’, ‘Immune regulation’, ‘Cell differentiation’, ‘Cell proliferation’, the known functions of Gata3 [19, 20]. Dataset GSE54932 is from a Setd2 knockout study, reporting 21 genes that are relevant to the phenotypic/functional differences between the wild type and the knockout. ‘Cell proliferation’, ‘DNA mismatch repair’, ‘Endodermal differentiation’, and ‘Histone modification’ were used as the contexts for the Setd2 knockout study since they are keywords representing well-known functions of Setd2 [24, 26]. Dataset GSE53398 of Barx2 knockout mice, was used for the last evaluation. The study reported that 47 genes significantly differs when Barx2 is silenced. For the corresponding knockout mice data, we used ‘Myoblast progeny’, ‘Muscle maintenance’, ‘Chondrogenesis’, ‘Morphogenesis’ as the contexts for CLIP-GENE [27–31].

#### Performance with the best context

In terms of F-measure, CLIP-GENE achieved better performance in finding phenotypical/functional relevant (validated) genes than GeneFriends, IA, and DEG method (Tables 1, 2 and 3), as well as prioritizing phenotypic/functionally relevant genes with proper ranks (Tables 4, 5 and 6). Context ‘Immune regulation’ achieved the best performance for the Gata3 knockout data, which performed about 5.4 times better than DEG, 2.4 better than IA, and 15 times better than GeneFriends while ranking 4 genes in the top 10 gene list among 25 validated genes. For the Setd2 knockout data, CLIP-GENE ranked 4 genes among 21 validated genes in top 10 list with the context ‘Endodermal differentiation’, achieving 11 times better than DEG, 6.7 times better than IA, and 72 times better than GeneFriends. For the Barx2 knockout data, context ‘Myoblast progeny’ achieved the best performance, achieving 4.8 times better than the DEG, 3.2 times better than IA method, and 9.7 times better than GeneFriends. In addition, CLIP-GENE was able to prioritize 2 genes among 47 validated genes in top 10 from Barx2 knockout data.

**Table 1:** Performance of CLIP-GENE while analyzing GSE47851 (Gata3 KO)

Methods	Precision	Recall	F-measure
DEG	0.0105	1	0.0208
IA	0.0239	0.72	0.0463
GeneFriends	0.0038	0.92	0.0075
CLIP-GENE (Immune regulation*)	**0.0613**	**0.64**	**0.1122**
CLIP-GENE (Inflammatory response)	0.0354	0.76	0.0677
CLIP-GENE (Cell differentiation)	0.0294	0.72	0.0564
CLIP-GENE (Cell proliferation)	0.0201	0.72	0.0391

The best performed measurement is marked with a star (*) with a bold text

**Table 2:** Performance of CLIP-GENE while analyzing GSE54932 (Setd2 KO)

Methods	Precision	Recall	F-measure
DEG	0.0099	0.5238	0.0195
IA	0.0183	0.1905	0.0333
GeneFriends	0.0015	0.5238	0.0031
CLIP-GENE (Endodermal differentiation*)	**0.2083**	**0.2381**	**0.2222**
CLIP-GENE (Cell proliferation)	0.0252	0.3333	0.0468
CLIP-GENE (DNA mismatch repair)	0.1304	0.1429	0.1364
CLIP-GENE (Histone modification)	0.0408	0.1905	0.0672

The best performed measurement is marked with a star (*) with a bold text

**Table 3:** Performance of CLIP-GENE while analyzing GSE53398 (Barx2 KO)

Methods	Precision	Recall	F-measure
DEG	0.0071	0.7872	0.0142
IA	0.0111	0.3617	0.0215
GeneFriends	0.0036	0.617	0.0071
CLIP-GENE (Myoblast progeny*)	**0.1818**	**0.0426**	**0.069**
CLIP-GENE (Muscle maintenance)	0.0476	0.0426	0.0449
CLIP-GENE (Chondrogensis)	0.1667	0.0426	0.0678
CLIP-GENE (Morphogenesis)	0.0217	0.4255	0.0412

The best performed measurement is marked with a star (*) with a bold text

**Table 4:** Gene prioritization results of CLIP-GENE at GSE47851 (Gata3 KO) data

	Context:	Context:	Context:	Context:
Reported gene	Immune regulation*	Inflammatory response	Cell differentiation	Cell proliferation
Relb	144	56	117	209
Nfkb2	-	-	-	-
Tnfrsf9	236	246	408	751
Tnfrsf21	-	-	-	-
Icos	16	44	**9**	66
Cysltr1	186	282	392	638
Kit	-	-	-	-
Il1r2	-	203	-	483
Il13	4	37	44	125
Il5	**5**	50	52	149
Areg	71	87	154	96
Il1rl1	67	109	237	507
Ccr8	108	88	281	643
Tph1	216	180	86	222
Htr1b	-	-	-	-
Cd244	-	-	-	-
Lta	19	**5**	122	203
Il10	**8**	**4**	47	74
Tnf	**1**	**1**	**1**	**2**
Nfkbia	-	33	341	563
Cdkn2b	78	256	255	122
Lif	-	-	-	402
Il2ra	84	206	88	546
Il9r	165	351	398	-
Il24	-	436	343	-
reproduced true positives/				
predicted candidates	16/260	19/536	18/613	18/896

The table represents how CLIP-GENE succeed to reproduce and prioritize the reported genes from the data produced study [19]. Genes that are ranked in top 10 are marked with bold font while star (*) represents best performed context

**Table 5:** Gene prioritization results of CLIP-GENE at GSE54932 (Setd2 KO) data

	Context:	Context:	Context:	Context:
Reported gene	Endodermal differentiation*	Cell Proliferation	DNA mismatch repair	Histone modification
Gata6	**3**	40	15	23
Sox7	**6**	72	-	-
Sox17	-	-	-	-
Dab2	16	44	-	65
Cubn	-	-	-	-
Cdx2	**7**	19	**5**	13
Psx1	-	-	-	-
Fgf5	-	-	-	-
Pax6	-	-	-	-
T	**2**	**2**	**1**	**2**
Gata4	-	-	-	-
Hnf1b	-	213	-	-
Colora1	-	-	-	-
Myo6	-	-	-	-
Pfn2	-	-	-	-
Cldn1	-	-	-	-
Vil1	-	-	-	-
Fgfr3	-	-	-	-
Fgfr4	-	28	-	-
Arc	-	-	-	-
Cd97	-	-	-	-
reproduced true positives/				
predicted candidates	5/24	7/278	3/23	4/98

The table represents how CLIP-GENE succeed to reproduce and prioritize the reported genes from the data produced study [24]. Genes that are ranked in top 10 are marked with bold font while star (*) represents best performed context

**Table 6:** Gene prioritization results of CLIP-GENE at GSE53398 (Barx2 KO) data

	Context:	Context:	Context:	Context:
Reported gene	Myoblast progeny*	Muscle maintenance	Chondrogensis	Morphogenesis
Gdnf	-	-	-	-
Id2	-	-	-	271
Mmp9	**9**	29	**8**	129
Smo	-	-	-	-
Sox2	-	-	-	**9**
Wisp1	-	-	-	-
Wisp2	-	-	-	-
Ahr	-	-	-	-
Axin2	-	-	-	-
Cacna2d3	-	-	-	-
Ccnd1	-	-	-	-
Ccnd2	-	-	-	-
Ctgf	-	-	-	-
Dlk1	-	-	-	319
Fgf7	-	-	-	-
Fst	-	-	-	307
Fzd7	-	-	-	-
Gdf5	-	-	-	-
Igf2	-	-	-	-
Klf5	-	-	-	96
Pdgfra	**4**	**8**	**4**	573
Pitx2	-	-	-	**4**
Tgfb3	-	-	-	780
Wnt5a	-	-	-	-
Fzd4	-	-	-	193
Fzd6	-	-	-	-
Sfrp1	-	-	-	188
Tle2	-	-	-	-
Dvl1	-	-	-	168
Nkd1	-	-	-	-
Porcn	-	-	-	367
Wif1	-	-	-	74
Wnt4	-	-	-	-
Axin1	-	-	-	-
Fstl1	-	-	-	-
Fstl3	-	-	-	-
Hey1	-	-	-	122
Hey2	-	-	-	-
Heyl	-	-	-	-
Hes1	-	-	-	34
Hes6	-	-	-	-
Snai1	-	-	-	295
Snai2	-	-	-	399
Snai3	-	-	-	526
Fos	-	-	-	-
Nrap	-	-	-	-
Id1	-	-	-	205
reproduced true positives/				
predicted candidates	2/11	2/42	2/12	20/923

The table represents how CLIP-GENE succeed to reproduce and prioritize the reported genes from the data produced study [25]. Genes that are ranked in top 10 are marked with bold font while star (*) represents best performed context

#### Performance with the worst context

In terms of F-measure, even with the worst performed context, CLIP-GENE achieved better performance in predicting phenotypic/functionally relevant genes. For the Gata3 knockout data, context ‘Cell proliferation’ performed 1.9 times better than DEG and 5.2 times better than GeneFriends, and slightly poor than IA. CLIP-GENE ranked one gene in the top 10 among 25 validated genes. The context ‘Cell proliferation’ performed the worst case for the Setd2 knockout data, which still performed better than DEG, IA, and GeneFriends while reporting one gene among 21 validated genes in top 10. ‘Morphogensis’ was the worst context for the Barx2 knockout dataset. However, CLIP-GENE still performs better than other methods while ranking 2 genes from the 47 validated genes in top 10, which again suggests that CLIP-GENE promises significant results than other compared methods even with the worst context.

### Performance comparison summary

The performance of CLIP-GENE depends on the context that the user provided. However, in terms of performance and prioritization, even with the context that performed worst, CLIP-GENE was consistently superior to DEG, IA, and GeneFriends.

## Discussion

Transcriptome data from mouse models with certain genes knocked out are widely used to investigate gene functions in terms of phenotypes. In order to determine genes that are affected by the knocked out TF, both selecting candidate genes and prioritizing genes are necessary. Only three tools are available for the mouse data, but none of these tools was appropriate to prioritize genes of user’s interest from knockout data. In this study, we present a novel web service that select and prioritize the candidate genes in terms of the user’s experimental context. Two major contributions are:

- CLIP-GENE allows researchers to specify the experimental conditions in a set of keywords. Our system automatically determines relevance between the keywords and genes so that we can provide rankings of the candidate genes in the userŠs context.
- CLIP-GENE provides a comprehensive web service for the mouse knockout experiments by integrating multiple resources into a single framework: mouse TF network, SNV information, PPI network, and literature information.

We believe that CLIP-gene will be useful for characterizing functions of TFs in mouse studies.

## Availability and requirements

**Project name:** CLIP-GENE**Project home page:** http://epigenomics.snu.ac.kr/CLIP-GENE **Requirements:** Internet Explorer, Chrome

## Materials

### Analyzing RNA-seq data: alignment to DEG calculation

We used mice RNA-seq dataset of GSE47851 [19], GSE54932 [24], and GSE53398 [25] that are retrieved from Gene Expression Omnibus (GEO) [32]. We used these three independent dataset to validate the performance of CLIP-GENE. Trim galore [33] was used for quality control while RSEM (v1.2.19) [34] is used for aligning reads to the mmu10 mouse reference genome. DEGs were analyzed by using EBSeq [35], a tool embedded in RSEM. Each tool was executed with a default option.

### TF network construction

The TF network describes the control mechanism of genes and it can be used as a blue print to understand the relationship between target genes and regulatory genes [36]. TF network is particularly useful when the knockout gene is TF. CLIP-GENE uses TF network to select candidate DEG genes by following edges between TF and target genes. TF network used for CLIP-GENE was constructed using normal inbred mice data that vary in strains, developmental stage, and tissues (150 samples of wild type mice RNA-seq data from 17 independent studies) [37–53]. NARROMI [54] was used for the TF network construction. Since NARROMI requires a transcription factor list and a gene list as input, we used a transcription factors list (including co-factors) from Animal Transcription Factor Database [55].

### Variant calling

Genome Analysis Tool Kit (GATK v3.3.0) [56] was used for calling variants from RNA-seq data. We performed GATK best practice workflows with default options. While processing the GATK RNA-seq pipeline, we used STAR (v2.4.0) [57] for aligning the reads, and Picard (v1.115) for marking the duplicates and sorting the reads.

### Biomedical entity search tool

BEST [17] API was utilized for calculating the relevance score of two different keywords. Performed 6th of July, 2016. Please note that relevance score could be calculated different due to the status of PubMed.

## Reviewers’ comments

### Reviewer 1: Dr. Sandor Pongor

**Summary** : The ms of Hur et al. describes a gene prioritization server designed for evaluating mouse knockout experiments. As the authors point out, general prioritization tools can not easily be used for mouse knockout data. The authors’ solution to the problem is to design a mouse-specific transcription factor network based on a variety heterogeneous data, and integrating it with another single nucleotide variant dataset. This extended network is used to prioritizing genes in a particular manner, taking in consideration the functional context.

**Recommendations**: This is a complex workflow which is not easily understood by the lay users, for instance it is not straightforward if a good performance is due the new data network, or the algorithm used by the server. In any case, the authors show that their prioritization method works better than other state/of/the art methodologies which were not explicitly designed for mouse experiments. The manuscript would benefit from the discussing some of the above issues, also it may me mentioned, if and to what extent the differences found between the various methods are statistically significant.

Authors’ response: *We highly appreciate the thoughtful comment. In order to determine whether the performance differences are due to the network, we performed additional experiments by excluding the network and also by utilizing other network.* *As a result, we found that CLIP-GENE has dependency to the network. CLIP-GENE performed better when the network information was utilized (Additional file* 1 *: Table S1) for Setd2 and Barx2 KO data. It was notable that the network did not decreased the recall rate. However, the adapted network was less effective for Gata3 KO data as it increased the number of false negatives (Additional file* 1 *: Table S1). In order to estimate the differences by network, we additionally tested CLIP-GENE with another network, RegNetwork [* 58 *], an integrative regulatory network that assembled regulatory information from multiple databases. When we used RegNetwork instead of our network, we found that CLIP-GENE with RegNetwork have increased the number of false negatives and the performance dropped with the best context while it performed better with the worst context on Gata3 and Setd2 KO data (Additional file* 1 *: Table S2). In addition, CLIP-GENE with RegNetwork generally performed better in Barx2 KO data, but the differences are small (Additional file* 1 *: Table S2).*

*In summary, we confirmed that network information is one of the major factor that benefits the precision of CLIP-GENE by rejecting many false positives for most of the data. However, it is considerable that recall decreases for certain data and the performance differs when different network is applied. Therefore, we have implemented CLIP-GENE (package version) so that advanced users can provide network topology as input.*

### Reviewer 2: Dr. Sanghyuk Lee

**Summary** : This manuscript describes a web server application for gene selection and prioritization for mouse TF knockout experiments. The flow of analysis pipeline is sound and several interesting ideas were implemented including (i) trimming out irrelevant genes using mouse TF network pre-calculated from massive mouse transcriptome data, (ii) gene ranking that reflects the biological contexts defined by user-supplied keywords, and (iii) gene prioritization using protein-protein interaction network. The application should be useful for analyzing mouse TF knockout experiments. Authors are recommended to address the following points to enhance the quality of manuscript.

**Recommendations** : The performance test was focused solely on F-measure which is a combined measure of precision and recall. Looking into the details in Tables 1, 2 and 3 shows that the precision of CLIP-GENE is far superior to others with lower recall rate regardless of context keywords. This is important because false negatives are the main problems for most users (molecular biologists or doctors). Adding a case with no keyword in CLIP-GENE would help readers estimate the extent of positive contribution from proper context words.

Authors’ response: *In order to estimate the performance when the context is not provided to CLIP-GENE, we excluded BEST during the analysis and ranked genes only with PPI shortest path information.*

*As a result, we found that CLIP-GENE generally performed better when the context was given (Additional file* 1 *: Table S3). Contexts was a major contributing factor on increasing the precision by rejecting high number of false positives. For example, in terms of F-measure, Setd2 KO data performed 8.8 times better with the best context, and 2.7 times better with the worst context. Also, we would like to emphasize that ranking genes without context (without BEST) is not effective. When we prioritize genes only with PPI shortest path information, a number of genes are prioritized with same ranks. This is because PPI-based ranking relies on the length of the shortest path. On a dense network such as PPI, it is natural that many nodes will have the same shortest path length. For instance, when BEST is not used, 210 genes among 1778 candidates was ranked as first and 1568 genes ranked as second for Gata3 KO data.*

Authors need to analyze the main reasons for the reduced recall rates of CLIP-GENE.

Authors’ response: *We determined that context was the major contributing factor for recall rates. As BEST finds the relationship between two different keywords on PubMed, BEST rejects candidate genes if the relationship of keywords is not on the literature. Therefore, the recall rate decreases when the context is inappropriate or the number of studies are few. It is noticeable that CLIP-GENE generally have low recall rate on Barx2 KO data than other datasets (Additional file* 1 *: Table S3), where BEST recognize 26 articles for Barx2 while others had more articles (1131 articles for Gata3, 109 articles for Setd2).*

The way to specify context seems to be limited. Many users would want to provide a list of keywords. For example, providing ‘immune regulation’ and ‘inflammatory response’ together should define better the molecular context of GATA3 KO mice. I am also curious about what the precision and recall rates would be for such combination of keywords.

Authors’ response: *We found that CLIP-GENE performs rather unpredictable when we give context with the combination of contexts (combining the best and worst performed context such as “Immune regulation cell proliferation”). The combination of contexts have slightly increased its performance of CLIP-GENE for Gata3 and Barx2, but decreased in Setd2 KO data (Additional file* 1 *: Table S3). Also, the combination of contexts have showed lower recall rate, indicating that the combination of contexts were found less in the literature than a single context.*

Authors need to explain the choice of context keywords for the SETD2 KO case. The most well-known functions of SETD2 are ‘histone modifications’ and ‘DNA mismatch repair’ in my opinion. But these two key words performed worse than ‘endodermal differentiation’.

Authors’ response: *We included more details into the manuscript to explain why this happened. We agree that ’histone modifications’ is one of the most well-known functions for Setd2. However, we would like to emphasize that the GSE54932 study mainly focused on the endodermal differences when Setd2 is silenced and reported genes that were related to them. As we used these reported genes as true positive, we believe that it is natural that the context of ’endodermal differentiation’ performed better than histone modification.*

The gene expression values are never used after the initial step of selecting DEGs. I guess that using gene expression in the prioritization step would help the performance of the program. This might be beyond the scope of current web server because expression values are not input data, but authors are recommended to give a brief review or comparison on such methods.

Authors’ response: *Thank you for sharing us your insightful thought. Currently, prioritizing genes with the combination of expression profile and mutiple data sources still remains a challanging task [* 8 *]. However, we do plan to use explicit expression profile during the prioritization process for the future release. We will continue to work on this important topic.*

## Additional file

Additional file 1
**Table S1.** Performance comparison of CLIP-GENE (excluding and including network) while analyzing Gata3, Setd2, and Barx2 knockout data. **Table S2.** Performance comparison of CLIP-GENE (applied network and RegNetwork) while analyzing Gata3, Setd2, and Barx2 knockout data. **Table S3.** Performance comparison of CLIP-GENE (no-context, best context, worst context, combination of context) while analyzing Gata3, Setd2, and Barx2 knockout data. (DOCX 20.4 kb)

## Abbreviations

DEG: Differentially expressed gene
GRN: Gene regulatory network
NER: Named entity recognition
PPI: Protein-protein interaction
SNV: Single nucleotide variant
TF: Transcription factor
