# Supplementary material for: CLIP-GENE: a web service of the condition specific context-laid integrative analysis for gene prioritization in mouse TF knockout experiments
Source: Biol Direct. 2016 Oct 24;11:57. doi: 10.1186/s13062-016-0158-x (PMC5078909; doi:10.1186/s13062-016-0158-x)
Supplement: Additional file 1 — Table S1. Performance comparison of CLIP-GENE (excluding and including network) while analyzing Gata3, Setd2, and Barx2 knockout data. Table S2. Performance comparison of CLIP-GENE (applied network and RegNetwork) while analyzing Gata3, Setd2, and Barx2 knockout data. Table S3. Performance comparison of CLIP-GENE (no-context, best context, worst context, combination of context) while analyzing Gata3, Setd2, and Barx2 knockout data. (DOCX 20.4 kb) [file 13062_2016_158_MOESM1_ESM.docx]

Table S1.

Performance comparison of CLIP-GENE (excluding and including network) while analyzing Gata3, Setd2, and Barx2 knockout data.

| Gata3 KO | Network excluded | | |
| --- | --- | --- | --- |
| Context | Precision | Recall | F-measure |
| Immune regulation | 0.0660 | 0.8 | 0.1219 |
| Cell proliferation | 0.0207 | 0.88 | 0.0405 |
|  | Network included | | |
| Immune regulation | 0.0613 | 0.64 | 0.1122 |
| Cell proliferation | 0.0201 | 0.72 | 0.0391 |
|  |  |  |  |
| Setd2 KO | Network excluded | | |
| Context | Precision | Recall | F-measure |
| Endodermal differentiation | 0.1667 | 0.2381 | 0.1961 |
| Histone modification | 0.0397 | 0.2381 | 0.0680 |
|  | Network included | | |
| Endodermal differentiation | 0.2083 | 0.2381 | 0.2222 |
| Histone modification | 0.0408 | 0.1905 | 0.0672 |
|  |  |  |  |
| Barx2 KO | Network excluded | | |
| Context | Precision | Recall | F-measure |
| Myoblast progeny | 0.1818 | 0.0426 | 0.0690 |
| Morphogenesis | 0.0210 | 0.4681 | 0.0403 |
|  | Network included | | |
| Myoblast progeny | 0.1818 | 0.0426 | 0.069 |
| Morphogenesis | 0.0217 | 0.4255 | 0.0412 |

Table S2.

Performance comparison of CLIP-GENE (applied network and RegNetwork) while analyzing Gata3, Setd2, and Barx2 knockout data.

| Gata3 KO | RegNetwork | | |
| --- | --- | --- | --- |
| Context | Precision | Recall | F-measure |
| Immune regulation | 0.0595 | 0.44 | 0.1048 |
| Cell proliferation | 0.0232 | 0.52 | 0.0444 |
|  | Applied network | | |
| Immune regulation | 0.0613 | 0.64 | 0.1122 |
| Cell proliferation | 0.0201 | 0.72 | 0.0391 |
|  |  |  |  |
| Setd2 KO | RegNetwork | | |
| Context | Precision | Recall | F-measure |
| Endodermal differentiation | 0.2353 | 0.1905 | 0.2105 |
| Histone modification | 0.0756 | 0.2381 | 0.1149 |
|  | Applied network | | |
| Endodermal differentiation | 0.2083 | 0.2381 | 0.2222 |
| Histone modification | 0.0408 | 0.1905 | 0.0672 |
|  |  |  |  |
| Barx2 KO | RegNetwork | | |
| Context | Precision | Recall | F-measure |
| Myoblast progeny | 0.2222 | 0.0426 | 0.0714 |
| Morphogenesis | 0.0227 | 0.4255 | 0.0431 |
|  | Applied network | | |
| Myoblast progeny | 0.1818 | 0.0426 | 0.069 |
| Morphogenesis | 0.0217 | 0.4255 | 0.0412 |

Table S3.

Performance comparison of CLIP-GENE (no-context, best context, worst context, combination of context) while analyzing Gata3, Setd2, and Barx2 knockout data.

| Gata3 KO |  | | |
| --- | --- | --- | --- |
| Context | Precision | Recall | F-measure |
| No context | 0.0107 | 0.76 | 0.0211 |
| Immune regulation | 0.0613 | 0.64 | 0.1122 |
| Cell proliferation | 0.0201 | 0.72 | 0.0391 |
| Immune regulation cell proliferation | 0.073 | 0.52 | 0.1281 |
|  |  |  |  |
| Setd2 KO |  | | |
| Context | Precision | Recall | F-measure |
| No context | 0.013 | 0.381 | 0.0252 |
| Endodermal differentiation | 0.2083 | 0.2381 | 0.2222 |
| Histone modification | 0.0408 | 0.1905 | 0.0672 |
| Endodermal differentiation Histone modification | 0.2 | 0.0952 | 0.1290 |
|  |  |  |  |
| Barx2 KO |  | | |
| Context | Precision | Recall | F-measure |
| No context | 0.0067 | 0.5319 | 0.0132 |
| Myoblast progeny | 0.1818 | 0.0426 | 0.069 |
| Morphogenesis | 0.0217 | 0.4255 | 0.0412 |
| Myoblast progeny Morphogenesis | 0.2 | 0.0426 | 0.0702 |
